# Subtipos del carcinoma luminal de mama según el consenso de Saint Gallen en un grupo de pacientes venezolanas

**DOI:** 10.7705/biomedica.5496

**Published:** 2021-09-22

**Authors:** Ángel Fernández-Tortolero, Aldo Reigosa-Yániz

**Affiliations:** 1 Centro de Investigaciones Médicas y Biotecnológicas, Facultad de Ciencias de la Salud, Universidad de Carabobo, Valencia, Venezuela Universidad de Carabobo Centro de Investigaciones Médicas y Biotecnológicas Facultad de Ciencias de la Salud Universidad de Carabobo Valencia Venezuela; 2 Departamento de Ciencias Fisiológicas, Escuela de Ciencias Biomédicas y Tecnológicas, Facultad de Ciencias de la Salud, Universidad de Carabobo, Valencia, Venezuela Universidad de Carabobo Departamento de Ciencias Fisiológicas, Escuela de Ciencias Biomédicas y Tecnológicas Facultad de Ciencias de la Salud Universidad de Carabobo Valencia Venezuela

**Keywords:** adenocarcinoma, neoplasias de la mama, Venezuela, Adenocarcinoma, breast neoplasms, Venezuela

## Abstract

**Introducción.:**

El cáncer de mama es la neoplasia maligna más frecuente en las mujeres de todo el mundo. Los distintos subtipos intrínsecos tienen pronósticos diferentes y su prevalencia varía significativamente según los criterios establecidos en el Consenso de Saint Gallen.

**Objetivo.:**

Clasificar los subtipos luminales del carcinoma de mama en una población de pacientes venezolanas según los consensos de Saint Gallen del 2009, 2011, 2013 y 2015.

**Materiales y métodos.:**

Se hizo un estudio retrospectivo en 209 pacientes con carcinoma ductal infiltrante de mama, atendidas en el Instituto de Oncología "Dr. Miguel Pérez Carreño" de Valencia, Venezuela.

**Resultados.:**

La distribución de los subtipos luminal A y B cambió después de reclasificar los casos según los consensos de 2011, 2013 y 2015; el subtipo luminal B fue el más común en la serie de estudio.

**Conclusiones.:**

Mediante la clasificación basada en los últimos criterios de Saint Gallen, se determinó un número mayor de tumores luminales B, lo que ayudaría a seleccionar a aquellas pacientes que no requieran la quimioterapia adyuvante y a quienes puedan beneficiarse de la terapia hormonal adyuvante en la práctica clínica.

## Abstract (en)

**Introduction:**

Breast cancer is the most frequent malignancy in women worldwide. Different intrinsic subtypes have different prognoses and their prevalence varies significantly according to the criteria established in the Saint Gallen Consensus.

**Objective:**

To classify the luminal subtypes of breast carcinoma according to Saint Gallen 2009, 2011, 2013, and 2015 consensuses in a group of Venezuelan patients.

**Materials and methods:**

We conducted a retrospective study in 209 patients with infiltrating ductal carcinoma of the breast followed up at the Institute of Oncology "Dr. Miguel Pérez Carreno" in Valencia, Venezuela.

**Results:**

The distribution of the luminal A and B subtypes changed after the reclassification of the cases according to 2011, 2013, and 2015 Saint Gallen Consensuses. The luminal B subtype was the most common in the study series.

**Conclusions:**

With the use of a classification based on the latest Saint Gallen criteria we identified more luminal B tumors. This could contribute to selecting those patients who can avoid adjuvant chemotherapy and/or benefit from the adjuvant hormonal therapy in clinical practice.

El carcinoma de mama es el cáncer más común en las mujeres a nivel mundial. En la práctica clínica se usa ampliamente la clasificación de los subtipos intrínsecos del carcinoma de mama determinados mediante inmunohistoquímica. Los tumores de mama poseedores de receptores de estrógeno (RE) o de progesterona (RP) se clasifican como luminal A o luminal B, y con expresión del receptor HER2 o sin ella. El subtipo luminal A se caracteriza por expresar gran cantidad de receptores de estrógeno y tener un pronóstico favorable, mejor que el del subtipo luminal B ^1^.

Los carcinomas luminales representan alrededor de dos tercios de todos los carcinomas de mama a nivel mundial. Este tipo de cáncer es muy heterogéneo e incluye perfiles de expresión génica y patrones de mutación cuyo curso clínico y respuestas al tratamiento sistémico son muy variadas. En la actualidad, se han propuesto varias terapias individualizadas según las características biológicas de cada subtipo luminal. Por lo tanto, la diferenciación de los tumores luminales conlleva importantes implicaciones terapéuticas ^2^.

La caracterización fenotípica de estos tumores se basa en los criterios del consenso de Saint Gallen, a partir del análisis de la expresión de RE, RP y HER2, y la medición del índice de proliferación (Ki-67), un marcador nuclear de la proliferación celular que se expresa en todas las fases del ciclo celular, excepto en la G0 ^3^. En el consenso de Saint Gallen del 2009, se enfatizó sobre la importancia de los marcadores de proliferación y se sugirieron para guiar la elección de la quimioterapia adyuvante en el tratamiento de pacientes con receptores hormonales positivos. Sin embargo, hay muchas limitaciones asociadas con la interpretación del marcador y las diferencias en los valores de corte, así como en los métodos de interpretación, la variabilidad entre observadores y la heterogeneidad de la expresión de Ki-67 ^4^^,^^5^.

Los resultados variables de la interpretación inmunohistoquímica de Ki-67 pueden deberse, principalmente, a la falta de consenso en torno a la metodología. Por esta razón, en el consenso de Saint Gallen del 2011 se sugirieron diferentes pautas para la diferenciación inmunohistoquímica de los carcinomas luminales A y B. El punto de corte de Ki-67 por debajo del 14 % fue el que mejor se correlacionó con la determinación de la expresión génica del subtipo luminal A. Por otra parte, se consideró un índice Ki-67 de 14 % o más para caracterizar el subtipo luminal B negativo para HER2 ^5^.

En el consenso de Saint Gallen del 2013, el panel de expertos recomendó que la distinción entre los tumores de subtipo luminal A y B podría mejorarse con la inclusión de una expresión del RP≥20 % como criterio para definir el subtipo luminal A. Asimismo, se eliminó el valor de umbral para el "Ki-67 alto" en la definición del subtipo luminal B. Se propuso un Ki-67 de 14 % o más para distinguir entre los tumores luminal B y los A, pero al considerar los estudios génicos, la mayoría del panel votó por un punto de corte de ≥20 %, aunque no lo recomendó explícitamente ^6^. En el último consenso de Saint Gallen, en el 2015, la mayoría del panel aceptó un umbral con un rango de 20 a 29 % para distinguir entre los subtipos luminal A y B ^3^.

Considerando que los carcinomas luminales de mama conforman al menos la mitad de todos los nuevos diagnósticos, determinar si un tumor es luminal A o B y cuál es la mejor forma de abordar el tratamiento, resulta clínicamente importante ^7^. Cabe destacar que el subtipo luminal A presenta una alta tasa de mejoría con el tratamiento hormonal y, en general, no se beneficia del uso de agentes quimioterapéuticos. Por otra parte, el tratamiento hormonal es menos efectivo en el subtipo luminal B, aunque estos tumores sí mejoran con la quimioterapia convencional y con aquella contra la HER2 ^2^^,^^4^.

En este contexto, en el presente estudio se analizó una serie de mujeres venezolanas con carcinoma de mama y se clasificaron los casos de acuerdo con las definiciones de los subtipos intrínsecos propuestas en los consensos internacionales de cáncer de mama de Saint Gallen, con el propósito de contribuir a identificar aquellas pacientes que podrían evitarse la quimioterapia adyuvante o beneficiarse de la terapia hormonal adyuvante, en la práctica clínica.

## Materiales y métodos

El estudio se llevó a cabo en mujeres atendidas en el Instituto de Oncología "Dr. Miguel Pérez Carreño" (IOMPC) de Valencia, Venezuela entre el 2014 y el 2016. Con la aprobación del Comité de Ética y de la Comisión de Investigación del IOMPC, se conformó una serie no aleatoria, de tipo intencional, de 209 pacientes con diagnóstico de carcinoma de mama con fenotipo luminal. Los tumores se clasificaron según los criterios de los consensos de Saint Gallen de 2009, 2011, 2013 y 2015 (cuadro 1).

**Cuadro 1:** Definición del subtipo luminal por inmunohistoquímica según los consensos de Saint Gallen

Consenso de Saint Gallen	Luminal A	Luminal B
2009	RE (+), RP (+) y HER2 (-)	RE (+), RP (+) y HER2 (+)
2011	RE (+), RP (+), HER2 (-) y Ki-67 (<14 %)	RE (+), RP (+), HER2 (+) y cualquier Ki-67 RE (+), RP (+), HER2 (-) y Ki-67 (≥14 %)
2013	RE (≥1 %), RP (>20 %), HER2 (-) y Ki-67 (<14 %)	RE (≥1 %), HER2 (+) y cualquier RP y Ki-67 RE (≥1 %), HER2 (-) y Ki-67 (≥14 %) o RP (<20 %)
2015	RE (≥1 %), RP (≥20 %), HER2 (-) y Ki-67 (<20 %)	RE (≥1 %), HER2 (+) y cualquier RP y Ki-67 RE (≥1 %), HER2 (-) y Ki-67 (≥20 %) o RP (<20 %)

RE: receptor de estrógeno; RP: receptor de progesterona; HER2: receptor del factor de crecimiento epidérmico humano 2

Los datos de interés para la investigación se tomaron de las historias clínicas, establecidas por el Servicio de Patología Mamaria del IOMPC. Para la supervivencia global en meses, se consideró como punto de corte un seguimiento de hasta 60 meses (5 años) y un mínimo de 36; la supervivencia se evaluó estableciendo el tiempo transcurrido desde el diagnóstico hasta la fecha de la muerte en caso de haber ocurrido antes de los 60 meses. En cuanto a los estudios de supervivencia y su relación con las variables clínico-patológicas y los marcadores inmunohistoquímicos, se tuvo en cuenta la definición de tumores luminales establecida en el consenso de Saint Gallen del 2009.

### Construcción de la matriz de tejidos

Las muestras tisulares se fijaron en formol y se incluyeron en parafina, siguiendo los métodos convencionales. De los bloques de parafina se obtuvieron secciones histológicas de 4 μm de espesor que, posteriormente, se tiñeron con hematoxilina y eosina. Se revisaron las preparaciones histológicas y se seleccionaron cuidadosamente las zonas con tumor, marcándolas en el bloque de parafina para construir las matrices de tejido según lo descrito en la literatura ^8^^,^^9^.

### Inmunohistoquímica

La 'desparafinación' de los cortes histológicos, su incubación con el anticuerpo primario (cuadro 2) y el posterior procesamiento de las muestras, se hicieron de acuerdo con lo establecido en investigaciones previas ^8^^,^^9^. Cada anticuerpo fue comparado con los controles adecuados, positivo o negativo, según lo establecido por la marca comercial. Para los receptores hormonales se consideró positivo cualquier tipo de inmunoexpresión, y se refirió el resultado como positivo o negativo para efectos del análisis estadístico. La expresión de la HER2 se evaluó según el patrón establecido en el estuche comercial Herceptest™.

**Cuadro 2:** Dilución, clon y fuente de anticuerpos utilizados

Marcador	Dilución	Clon	Marca comercial	Localización del marcaje
RE	1/50	GF 11	Novocastra	Nuclear
RP	1/100	636	Dako	Nuclear
HER2	Prediluido	Herceptest	Dako	Membrana
Ki-67	1/100	MIB-1	Dako	Nuclear

RE: receptor de estrógeno; RP: receptor de progesterona; HER2: receptor del factor de crecimiento epidérmico humano 2

Para la cuantificación del Ki-67 se tomaron cuatro microfotografías de cada caso, dos de cada cilindro, en un microscopio Zeiss Axiostar plus con cámara Canon incorporada y conectada al ordenador con el programa Axiovision. Luego se contaron los núcleos positivos y negativos en cada imagen, utilizando el programa Bronce, diseñado por el ingeniero Víctor Barrios de la Universidad de Carabobo. Se sumaron las cifras de los cuatro conteos y se obtuvo el índice de proliferación como promedio del porcentaje de positividad en cada caso.

### Análisis estadístico

Los datos se analizaron mediante el paquete estadístico SPSS™, versión 22. La asociación entre las variables se analizó con la prueba de ji al cuadrado y el test exacto de Fisher. El estudio de supervivencia se hizo mediante el método de Kaplan-Meier y se probó utilizando la prueba de *log-rank.* En los análisis multivariantes, se empleó el modelo de riesgo proporcional de Cox. Se consideraron significativos los valores de p<0,05.

## Resultados

La edad media de las pacientes en el momento del diagnóstico era de 51,6 años. El estadio clínico más frecuente fue el III e, histológicamente, la mayoría de los tumores fue moderadamente diferenciada. La mayoría de las pacientes continuaba con vida al final del seguimiento. Los principales datos clínico-patológicos de las pacientes incluidas en este estudio se detallan en el cuadro 3.

**Cuadro 3:** Características clínico-patológicas de la serie

Variable		n	(%)
Edad (años): media (rango)	---	51,6	(27-85)
Número total de casos	---	209	(100)
Edad en años	<50	84	(40,2)
	≥50	125	(59,8)
Estadio clínico	I	6	(2,9)
	II	84	(40,2)
	III	110	(52,6)
	IV	9	(4,3)
Grado histológico	I	49	(23,4)
	II	121	(57,9)
	III	39	(18,7)
RP	<20 %	57	(27,3)
	≥20 %	152	(72,7)
HER2	Negativo	182	(87,1)
	Positivo	27	(12,9)
Ki-67	<14 %	74	(35,4)
	14-19 %	37	(17,7)
	≥20 %	98	(46,9)
Supervivencia global (meses)	---	50,1	
Estado	Viva	109	(52,2)
	Fallecida	100	(47,8)

RE: receptor de estrógeno; RP: receptor de progesterona; HER2: receptor del factor de crecimiento epidérmico humano 2

Las distribuciones de los subtipos luminal A y B cambiaron según lo establecido en los consensos de Saint Gallen. Con base en los criterios del 2009, los tumores luminales A fueron los más comunes. Sin embargo, después de la reclasificación de los casos según los consensos del 2011, 2013 y 2015, la proporción de los tumores del subtipo luminal A disminuyó drásticamente, en tanto que la de los de subtipo luminal B tuvo un aumento significativo (cuadro 4). A pesar de ello, en el estudio de supervivencia se evidenció que el subtipo luminal A se relacionó con una mayor supervivencia global que el luminal B, independientemente del criterio de clasificación, pero con significación estadística según lo establecido en los consensos de Saint Gallen del 2011, 2013 y 2015 (cuadro 5).

**Cuadro 4:** Distribución del subtipo luminal según lo establecido en los consensos de Saint Gallen

Consenso de Saint Gallen	Subtipo molecular	
	Luminal A	Luminal B
	n (%)	n (%)
2009	152 (72,7)	57 (27,3)
2011	61 (29,2)	148 (70,8)
2013	56 (26,8)	153 (73,2)
2015	78 (37,3)	131 (62,7)

**Cuadro 5:** Cambios en la supervivencia global de pacientes con tumores luminales según lo establecido en los consensos de Saint Gallen

Consenso Saint Gallen	Supervivencia global (media de meses ± DE)		p
	Luminal A	Luminal B	
2009	51,0 ± 1,9	49,7 ± 1,1	0,227
2011	57,9 ± 0,9	46,9 ± 1,2	<0,001
2013	58,1 ± 0,9	47,1 ± 1,2	<0,001
2015	57,1 ± 0,9	45,9 ± 1,3	<0,001

DE: desviación estándar

Para verificar si las modificaciones individuales habían mejorado la clasificación inicial de los tumores, en los siguientes análisis se tuvieron en cuenta los criterios de clasificación del subtipo luminal del consenso de Saint Gallen del 2009. Se evaluó la categorización de las pacientes en función de la supervivencia global con los valores de Ki-67 (<20 % Vs. ≥20 %) y receptores de progesterona (<20 % Vs. ≥20 %) en los tumores luminales A y, además, el estado de la HER2 en los casos del subtipo luminal B. Solo el Ki-67 (luminales A y B) y la HER2 (luminal B) tuvieron significación estadística en la subclasificación de los tumores (cuadro 6), con un valor independiente del resto de las variables clínico-patológicas valoradas (cuadro 7).

**Cuadro 6:** Supervivencia global de pacientes con tumores luminales según lo establecido en el consenso de Saint Gallen del 2009 y en relación con los marcadores inmunohistoquímicos

Marcador		Supervivencia global (media de meses ± DE)			
		Luminal A	p	Luminal B	p
Ki-67	<20	56,6 ± 1,1	<0,001	54,7 ± 1,6	0,019
	≥20	44,0 ± 1,7		40,8 ± 3,2	
RP	<20	48,9 ± 3,8	0,780	48,0 ± 2,5	0,735
	≥20	51,5 ± 1,1		45,3 ± 3,8	
HER2	Negativo	---	---	50,1 ± 2,6	0,015
	Positivo	---		42,8 ± 3,3	

RP: receptor de progesterona; DE: desviación estándar; HER2: receptor del factor de crecimiento epidérmico humano 2

**Cuadro 7:** Análisis multivariante de la supervivencia global de pacientes con tumores luminales establecido en el consenso de Saint Gallen del 2009, y variables clínico-patológicas y moléculas estudiadas

Variable	Supervivencia global (media de meses ± DE)			p
	Luminal A CR (IC al 95 %)	p	Luminal B CR (IC al 95 %)	
Ki-67 20 %	4,487 (2,619-7,687)	<0,001	3,172 (1,445-6,964)	0,004
RP 20 %	1,221 (0,572-2,606)	0,605	0,975 (0,0446-2,133)	0,950
HER2	---	---	2,428 (1,136-5,188)	0,022
Edad	0,842 (0,520-1,364)	0,485	0,909 (0,436-1,895)	0,799
GH	1,263 (0,828-1,927)	0,279	2,246 (1,192-4,232)	0,012
EC	1,951 (1,196-3,183)	0,007	4,537 (1,962-10,492)	<0,001

CR: cociente de riesgo; IC: intervalo de confianza; RP: receptor de progesterona; HER2: receptor del factor de crecimiento epidérmico humano 2; DE: desviación estándar; GH: grado histológico; EC: estadio clínico

**Figura 1:**
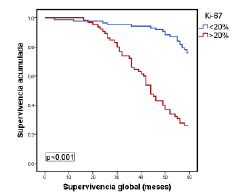
Relación entre la supervivencia global y la de pacientes con el subtipo luminal A, según el consenso de Saint Gallen del 2009 y el porcentaje de expresión de Ki-67 con el punto de corte en 20 %

**Figura 2:**
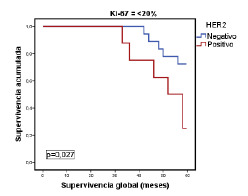
Relación entre la supervivencia global y la de pacientes con el subtipo luminal B, según el consenso de Saint Gallen del 2009, el estado de HER2 y un porcentaje de expresión de Ki-67 de <20%

**Figura 3:**
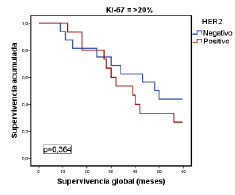
Relación entre la supervivencia global y la de pacientes con el subtipo luminal B, según el consenso de Saint Gallen del 2009, el estado de HER2 y un porcentaje de expresión de Ki-67 de >20 %

Por último, en la figura 1 se muestra la relación entre la supervivencia global y los tumores del subtipo luminal A, según el porcentaje de expresión del Ki-67 con el punto de corte en 20 %. Por su parte, en la figura 2 se evidencia la relación estadísticamente significativa entre la supervivencia global y los tumores del subtipo luminal B (negativos o positivos para HER2), según si el porcentaje de expresión de Ki-67 es menor o mayor del 20 %, pero sin significación estadística (figura 3).

## Discusión

En el presente estudio, se observó que el fenotipo molecular predominante fue el luminal A frente al luminal B, según los criterios previos al consenso de Saint Gallen del 2011. Este predominio ha sido reportado ampliamente en la literatura médica, aunque con discrepancias en los porcentajes que representan cada subtipo molecular; posiblemente, esto se deba al método de clasificación utilizado, pues en varios de los estudios solo se consideraron como luminal B los casos que coexpresaban receptores hormonales y la HER2, o, también, al tipo de población incluida en dichos estudios ^9^^,^^10^. En este sentido, en un estudio se evidenció que los hispanos y latinos tenían una prevalencia relativamente baja de tumores del subtipo luminal A comparados con los afroamericanos, y un aumento de las tasas de los tumores del subtipo luminal B y positivos para HER2 ^11^.

En consonancia con lo expuesto y después de la reclasificación de los tumores de este estudio, el subtipo luminal B fue el más común según los criterios de los consensos de Saint Gallen del 2011, 2013 y 2015. En dos estudios recientes se encontró una mayor prevalencia del subtipo luminal B en mujeres europeas (57,1 %) y chinas (68,5 %), al clasificar el cáncer de mama siguiendo las recomendaciones de Saint Gallen del 2013 ^6^^,^^12^. En otros estudios en Italia, se registraron hallazgos similares (34 % de luminal A y 36 % de luminal B), así como en Arabia Saudita (3,9 % de luminal A y 16 % de luminal B) ^13^^,^^14^. En Latinoamérica, Gómez, *et al.*^15^ reportaron en su estudio que el luminal B representaba más del 50 % de los subtipos intrínsecos y Serrano-Gómez, *et al.*^16^ informaron una frecuencia de 26,2 % para el luminal A y de 37,2 % para el luminal B.

Asimismo, los estudios de supervivencia revelan que la clasificación basada en los consensos de Saint Gallen se relaciona con la biología del tumor, pues ambos subtipos moleculares mostraron patrones de supervivencia similares a los descritos en los estudios pioneros de Perou y Sorlie ^17^^-^^19^, validados por la gran mayoría de las publicaciones posteriores. En este estudio, el subtipo molecular luminal A presentó un mejor pronóstico frente al luminal B, con una diferencia estadísticamente significativa según lo establecido en las reuniones de expertos del 2011 al 2015 y en consonancia con otros estudios previos ^15^^,^^16^.

Es importante destacar que la aplicación de los criterios de Saint Gallen del 2013 en este estudio, y en otros, resultó en una mayor frecuencia de tumores del subtipo luminal B después de emplear un valor de corte para el índice de proliferación Ki-67 de ≥14 % y una expresión de RP de <20 % en el subtipo luminal B negativo para HER2. En la serie se encontraron dos grandes factores de discriminación del valor de pronóstico de la evolución. El índice de proliferación ya se ha reportado como un importante factor de pronóstico ^20^^,^^21^, tanto así que, en estudios previos, ya se había sugerido que aquellos casos con un índice mayor a 14 % debían clasificarse como luminal B en lugar de luminal A ^21^^,^^22^. Se observaron diferencias significativas en la evolución, tanto con un punto de corte de 14 % como con uno de 20 %. En relación con el luminal A, se establecieron dos subgrupos con significado pronóstico independiente: el grupo con expresión únicamente de RE y RP y Ki-67 <20 % presentaba un mejor pronóstico, y los grupos de luminal A con Ki-67 ≥20 %, un peor pronóstico.

Por su parte, en el subtipo luminal B, la expresión del Ki-67 también demostró tener un valor pronóstico independiente con el punto de corte en 20 %, similar a lo reportado en otro estudio ^23^. Los diferentes subtipos de esta clase, establecidos según la expresión de HER2, también mostraron diferencias estadísticamente significativas en la supervivencia a los cinco años, resultados similares a los de otros autores que han reportado un peor pronóstico para los casos con expresión de HER2, aunque independientemente del estado del Ki-67 ^22^^,^^24^^-^^26^.

Asimismo, en la reunión del consenso de Saint Gallen del 2013, se agregó la expresión de PR con el punto de corte en 20 % para distinguir entre los tumores de subtipo luminal A y luminal B negativos para HER2. A este respecto, en otro estudio se encontró que la expresión de receptores de progesterona era significativamente mayor en el subtipo luminal A y que la presencia de más del 20 % de células tumorales poseedoras de estos receptores tenía un valor estadísticamente significativo para predecir diferencias en la supervivencia de los pacientes con este subgrupo de tumores ^27^. En los carcinomas de subtipo luminal B, autores como Konecny, *et al.*^28^, y Kim, *et al.*^29^, han informado hallazgos similares, los cuales difieren de lo encontrado en el presente estudio, en el que la expresión de receptores de progesterona con el punto de corte en 20 % no registró diferencias estadísticamente significativas en la supervivencia de las mujeres incluidas en la serie.

En resumen, los hallazgos del estudio indican que el uso de una clasificación basada en los criterios de Saint Gallen del 2013, y no exclusivamente en los marcadores RE, RP y HER2, de pacientes venezolanas, permitió clasificar más tumores del subtipo luminal B, los cuales están asociados con un mal pronóstico, lo que permitiría que se beneficiaran de un tratamiento más agresivo para reducir la probabilidad de recurrencias. Si la gran prevalencia de tumores del subtipo luminal B es una característica intrínseca de nuestra población, el desarrollo de esquemas quimioterapéuticos específicos para estas pacientes mejoraría su supervivencia.

Estos hallazgos deben interpretarse en el contexto de las limitaciones del estudio, entre ellas, el hecho de que las pacientes eran tratadas en un solo centro de salud (IOMPC) en Venezuela, así como el tamaño de la muestra considerada para los análisis de supervivencia global.
